# Feasibility of linac-based fractionated stereotactic radiotherapy and stereotactic radiosurgery for patients with up to ten brain metastases

**DOI:** 10.1186/s13014-022-02185-1

**Published:** 2022-12-28

**Authors:** Masanori Hirata, Kazuaki Yasui, Naofumi Oota, Hirofumi Ogawa, Tsuyoshi Onoe, Sayo Maki, Yusuke Ito, Kenji Hayashi, Hirofumi Asakura, Shigeyuki Murayama, Koichi Mitsuya, Shoichi Deguchi, Katsumasa Nakamura, Nakamasa Hayashi, Tetsuo Nishimura, Hideyuki Harada

**Affiliations:** 1 Radiation and Proton Therapy Center, Shizuoka Cancer Center, 1007 Shimonagakubo, Nagaizumi-Cho, Sunto-Gun, Shizuoka, 411-8777 Japan; 2 Division of Neurosurgery, Shizuoka Cancer Center, Shizuoka, Japan; 3 Department of Radiation Oncology, Hamamatsu University Hospital, Shizuoka, Japan

**Keywords:** Brain metastases, Linac-based fractionated stereotactic radiotherapy, Linac-based stereotactic radiosurgery, Overall survival, Local control, Radiation necrosis

## Abstract

**Background:**

Linac-based fractionated stereotactic radiotherapy (fSRT) and stereotactic radiosurgery (SRS) are increasingly being used to manage patients with multiple metastases. This retrospective cohort study aimed to compare the outcomes after linac-based fSRT and SRS between three patient groups classified based on the number of brain metastases (BMs): 1 BM, 2–4 BM, 5–10 BM.

**Methods:**

The data of consecutive patients with 1–10 BMs treated with fSRT or SRS between July 2016 and June 2018 at a single institution were collected. Patients with previous whole-brain radiotherapy (WBRT), concurrent use of WBRT, or surgical resection were excluded from the analysis. A total of 176 patients were classified into three groups according to the number of BMs: 78, 67, and 31 patients in 1 BM, 2–4 BM, and 5–10 BM, respectively. The Kaplan–Meier method was used to estimate overall survival (OS) curves, and the cumulative incidence with competing risks was used to estimate local control (LC), distant intracranial failure (DIF), and radiation necrosis (RN).

**Results:**

Median OS was 19.8 months (95% confidence interval [CI] 10.2–27.5), 7.3 months (4.9–11.1), and 5.1 months (4.0–9.0) in 1 BM, 2–4 BM, and 5–10 BM, respectively. Compared to 2–4 BM, 1 BM had significantly better OS (hazard ratio [HR] 0.59, 95% CI 0.40–0.87; *p* = 0.0075); however, 5–10 BM had comparable OS (HR 1.36, 95% CI 0.85–2.19; *p* = 0.199). There was no significant difference in LC, DIF, and RN between tumor number groups, but DIF was lower in 1 BM. RN of grade 2 or higher occurred in 21 patients (13.5%); grade 4 and 5 RN were not observed.

**Conclusions:**

The linac-based fSRT and SRS for patients with 5–10 BMs is comparable to that for patients with 2–4 BMs in OS, LC, DIF, and RN. It seems reasonable to use linac-based fSRT and SRS in patients with 5–10 BMs.

## Background

The use of fractionated stereotactic radiotherapy (fSRT) and stereotactic radiosurgery (SRS) are broadly indicated for up to 4 brain metastases (BMs) based on large phase III trials that randomized patients with limited BMs to SRS with or without whole-brain radiation therapy (WBRT) [1]. Previous gamma knife SRS study by Yamamoto et al. (JLGK 0901) showed that overall survival (OS) and safety for patients with 5 to 10 BMs and those with 2 to 4 BMs were comparable [2]. A gamma knife SRS study by Hughes et al. [3] also demonstrated that there was no significant difference in OS between patients with 2 to 4 BMs and those with 5 to 15 BMs. These studies imply that SRS can be considered in patients with ≥ 5 BMs.

However, in the JLGK 0901 study, tumors with a maximum diameter > 3 cm, a maximum tumor volume > 10 cm^3^, and a total volume of 15 cm^3^ were excluded [2]. In a multi-institutional and retrospective study by Hughes et al. [3], there was no information on tumor size. At our institution, in the case of BMs with up to 10 lesions, fSRT is indicated even for tumors > 3 cm in appropriate fractionations.

However, since several other studies have shown that tumor volume is more correlated with OS than the number of BMs [4–9], it is still unclear whether OS is comparable between patients with 5–10 BMs and those with 2–4 BMs when including tumors larger than 3 cm. In addition to the size of tumors, there is the question of whether the outcomes are the same with gamma knife SRS and linac-based SRS. Tuleasca et al. [10] and Sebastian et al. [11] reported that the OS of BMs treated with gamma knife SRS and linac-based SRS was the same. However, since both studies were mostly based on a limited number of lesions, it is uncertain whether the same results can be obtained for 5 to 10 BMs. For the reasons stated above, it is still arguable whether the implication of the gamma knife SRS study should also be true for up to 10 BMs, including larger tumors with linac-based fSRT and SRS.

In this study, we retrospectively reviewed the outcomes of linac-based fSRT and SRS for patients with up to 10 BMs, classified based on the number of BMs: Group A, 1 tumor; Group B, 2–4 tumors; and Group C, 5–10 tumors.

## Methods

### Patients

This study was approved by the institutional ethics committee, and an opt-out method for informed consent was granted, considering the retrospective nature of this analysis. This retrospective cohort study was conducted in a single institution. The medical records of patients with 1–10 BMs treated with linac-based fSRT or SRS between July 2016 and June 2018 at our institution were reviewed. Patients under 18 years of age, patients who had undergone prior WBRT or WBRT within 30 days after fSRT or SRS, or patients who underwent surgical resection before fSRT or SRS were excluded from the analysis. Survival information was obtained from the clinical follow-up data. Patient and tumor clinicopathological characteristics were extracted from the medical records, including age at treatment, sex, primary sites (grouped by lung cancer, gastrointestinal cancer (GI), breast cancer, and others), recursive partitioning analysis (RPA; Grade 1 to 3), Karnofsky performance status (KPS; ≥ 80 or ≤ 70), systemic therapy administered after fSRT or SRS, and volume data of BMs. Although DS-GPA has been used as a prognostic indicator of BMs in some specific malignant diseases [12], we adopted RPA in our study as in the JLGK 0901 study [2] because our study assessed a variety of malignant diseases. We excluded patients who did not come to the hospital after fSRT and SRS due to relocation or death. Shortly after treatment, follow-up magnetic resonance imaging (MRI) was performed 1–2 months after treatment and every 3–6 months after that as appropriate to evaluate post-treatment effects and side effects.

### Treatment

The treatment planning computed tomography (CT) taken before fSRT and SRS and fused with contrast-enhanced T1-weighted images were used to draw the gross tumor volume (GTV) as well as normal brain tissue and risk organs such as the brainstem and optic nerves.

For treatment, planning target volume (PTV) is assumed to have a margin of 1.5 mm in all directions for the GTV. fSRT was chosen when the target lesion was large, greater than approximately 2 cm in diameter, or target lesion located in eloquent areas (brainstem, thalamus, or basal ganglia), or located close to the optic nerves or chiasm. Prescribed doses were, in principle, 15–24 Gy for SRS and 30–35 Gy in five fractions for fSRT and were designed so that the prescribed dose would cover at least 95% of the PTV. In some patients, the prescribed dose was adjusted appropriately, considering the tumor volume, tumor localization, and consequently the dose/volume data on normal tissue. Based on previous studies [13, 14], the dose constraints for fSRT and SRS are as follows: the brain volume receiving 20 Gy (V_20-Gy_) is less than 23 cc in fSRT while that receiving 12 Gy (V_12-Gy_) is less than 10 cc in SRS. TrueBeam STx (Varian, Palo Alto, California, USA) was used as the treatment device. We introduced a radiation therapy planning software named Elements Multiple Brain Mets SRS (BrainLAB, Munich, Germany), a single-isocenter and dynamic conformal arc therapy that can irradiate multiple lesions simultaneously. Multiple brain Mets SRS was selected in most patients (168 out of 176) unless single isocenter irradiation would be inappropriate for target locations. For these patients, iPlan (BrainLAB, Munich, Germany) was applied instead of multiple brain Mets SRS, which requires isocenters for each tumor.

### Statistical analysis

The study endpoints included OS rate, local control (LC), distant intracranial failure (DIF), and radiation necrosis (RN). Patients were grouped according to their clinical information, and outcomes were analyzed. Patient characteristics were compared using the chi-square test or Fisher's exact test for categorical outcomes, as appropriate. For non-parametric ordinal variables, Wilcoxon rank-sum test was applied between two groups, and the Kruskal–Wallis test was applied for three or more groups.

OS was defined as the time from the date of treatment initiation to death. Local recurrence was defined as a continuous increase in the size of contrast-enhanced T1-weighted lesions on at least two consecutive post-treatment follow-up MRI scans. DIF was indicated by a post-treatment follow-up MRI showing the appearance of new lesions. RN was indicated by an increase in contrast-enhanced lesions at the irradiated site alongside edematous lesions in the surrounding brain tissue on follow-up MRI and/or clinical diagnosis by neurosurgeons. If it was difficult to distinguish between brain necrosis or recurrence after treatment, we also determined whether there was an increase in the relative cerebral blood volume ratio (rCBV) on perfusion CT and those with poorer rCBV were defined as RN [15]. The grade of RN was based on the Common Terminology Criteria for Adverse Events v 5.0. When multiple BMs were subjected to irradiation, even if one of them appeared as a recurrence or RN, it was considered an event.

Survival curves were generated using the Kaplan–Meier method. OS was censored on the last follow-up date, while LC, DIF, and RN were censored on the last imaging date. Cox proportional hazards analysis was used in both univariate and multivariate analyses to determine the covariates that predicted OS. Factors with overlapping content, such as RPA encompassing information on age, were excluded from the analysis. P value of < 0.01 was considered significantly different. For the analysis of LC, DIF and RN, the Fine-Gray method was used for competing risk cumulative incidence analysis, with death defined as a competing risk. The factors known from previous studies to be associated with treatment outcomes were included in the analysis [1, 2]. Since our study included differing prescribed dose fractions and number of BMs in fSRT and SRS, the characteristic of the dose-volume to the normal brain was not selected as one of the factors in the RN analysis.

In the analysis, the factors were divided as follows:

The number of BMs (patients with one tumor: Group A, patients with two to four tumors: Group B, patients with five to ten tumors: Group C), age (< 65 years old or ≥ 65 years), sex, primary tumor sites (lung cancer, GI, breast cancer, other cancers), KPS (≥ 80 or ≤ 70), the status of control of extracranial lesions (controlled or uncontrolled), irradiation method (SRS or fSRT), and total PTV volume (Group 1; ≥ 15 cm [3], Group 2; ≥ 4, < 15 cm [3], Group 3; < 4 cm [3]). The maximum tumor volume of the BMs was excluded from this analysis because it had a high correlation coefficient with the total tumor volume. Systemic treatment was excluded as a factor in this study especially when either the patient group had various malignant diseases, the presence or absence of systemic therapy was not a prognostic indicator in a prior prospective trial [1] or not added to the analysis. [2]

All statistical analyses were performed using EZR software (Saitama Medical Center, Jichii Medical University, Saitama, Japan). [16]

## Results

### Patient characteristics

**Fig. 1:**
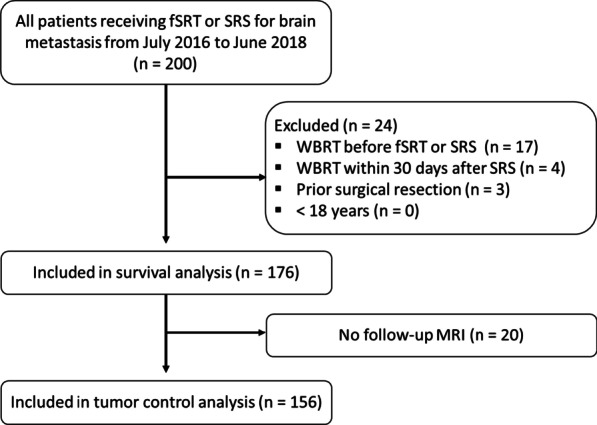
Flowchart of patient inclusion. fSRT, fractionated stereotactic radiotherapy; *SRS* Stereotactic radiosurgery, *WBRT* Whole brain radiotherapy

All patients who underwent fSRT or SRS for BMs between July 2016 and June 2018 (*n* = 200) were enrolled, and 176 patients with BMs who met the study inclusion criteria were analyzed for survival. Of these patients, 156 who underwent follow-up MRI were included in the analysis of LC, DIF, and RN (Fig. 1).

The median observation period for all patients was 8.9 months (range 0.1 to 36.5 months); the median observation period for surviving patients was 21.4 months (range 1.2–34.8 months). The median age of patients was 69.5 years (19–90 years), and lung cancer (111 patients, 63.1%) was the most common primary lesion. The group with 1 tumor (group A) included 78 patients (44.3%), group B (2–4) had 67 patients (38.1%), and group C (5–10) had 31 patients (17.6%). Regarding irradiation method, 97 patients (55%) received SRS, and 79 patients (45%) received fSRT. The median total PTV volume was 6.03 cm [3], and the mean total PTV volume was 11.22 cm^3^ (0.296–79.025 cm^3^). Regarding total PTV of all patients, 42 patients (24%) had a total PTV ≥ 15 cm^3^, 68 patients (39%) had ≥ 4, < 15 cm^3^, and 66 patients (37%) had < 4 cm^3^ (Table 1).

**Table 1:** Patient characteristics (*n* = 176)

	All cases (*n* = 176)	Group A: one tumor (*n* = 78)	Group B: two to four tumors (*n* = 67)	Group C: five to ten tumors (*n* = 31)	*p* value
*Age (years)*					0.156
Median	69.5	69.6	71.3	68.5	
(range)	(19–90)	(23–88)	(19–90)	(21–82)	
< 65 (%)	49 (27.8)	25 (32.1)	13 (19.4)	11 (35.5)	
≥ 65 (%)	127 (72.2)	53 (67.9)	54 (80.6)	20 (64.5)	
Gender					0.73
Male	97(55%)	43(55%)	35(52%)	19(61%)	
Female	79(45%)	35(45%)	32(48%)	12(39%)	
*Primary sites*					0.952
Lung	111(63%)	48(61.5%)	45(67%)	18(58%)	
GI	20(11%)	9(11.5%)	6(9%)	5(16%)	
Breast	8(5%)	4(5%)	3(4%)	1(3%)	
Others	37(21%)	17(22%)	13(20%)	7(23%)	
*KPS*					0.604
≥ 80	96(55%)	46(59%)	34(51%)	16(52%)	
≤ 70	80(45%)	32(41%)	33(49%)	15(48%)	
RPA					0.24
12	11(6%)	6(8%)	2(3%)	3(10%)	
3	119(68%)	56(72%)	46(69%)	17(55%)	
	46(26%)	16(20%)	19(28%)	11(35%)	
*Extracranial*					0.124
*Lesions*					
Controlled	50(28%)	27(35%)	18(27%)	5(16%)	
Uncontrolled	126(72%)	51(65%)	49(73%)	26(84%)	
fSRT or SRS					0.846
SRS	97(55%)	44(56%)	35(52%)	18(58%)	
fSRT	79(45%)	34(44%)	32(48%)	13(42%)	
*Systemic*					0.55
*Therapy*					
Yes	44(56%)	42(63%)	16(52%)	102(58%)	
No	34(44%)	25(37%)	15(48%)	74(42%)	
*Total PTV*					0.022
*volume (cm*^3^)					
1 (≥ 15)	42(24%)	11(14%)	19(28%)	12(39%)	
2 (≥ 4, < 15)	68(39%)	33(42%)	22(33%)	13(42%)	
3 (< 4)	66(37%)	34(44%)	26(39%)	6(19%)	

*G*I Gastrointestinal cancers, *KPS* Karnofsky performance status, *RPA* Recursive partitioning analysis, *fSRT* Fractionated stereotactic radiotherapy, *SRS* Stereotactic radiosurgery *PTV* Planning target volume

### Survival and tumor control

**Fig. 2:**
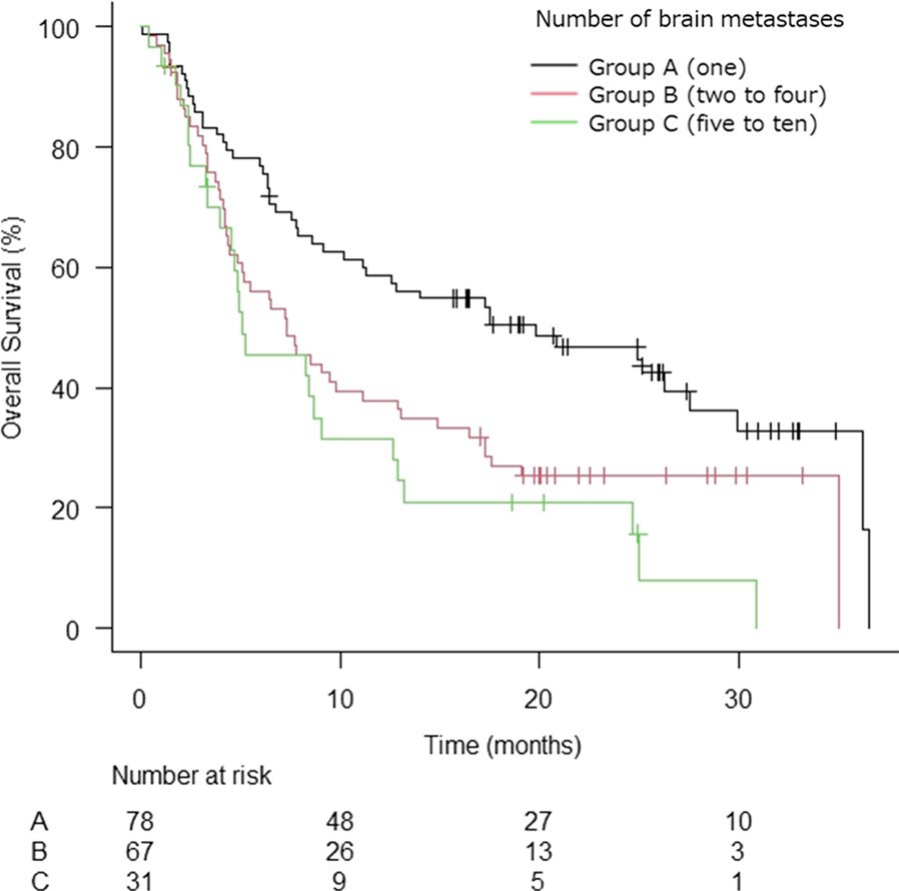
Kaplan–Meier curve of overall survival rate of all patients analyzed by tumor number (*n* = 176)

The Kaplan–Meier curve shows the OS rate in all patients analyzed by tumor number (Fig. 2).

The median survival time was 9.2 months (95% confidence interval [CI] 7.3 to 13.1 months), and 1-year and 2-year OS were 46.2% and 34.1%. The median survival was 19.8 months for tumor group A (95% CI 10.2–27.5; p < 0.001), 7.3 months for group B (95% CI 4.4–11.1), and 5.1 months for group C (95% CI 4.0–0.7). Of the 123 confirmed deaths, there were 26 (21.1%) neurological deaths.

Table 2 shows the analysis of OS and clinical factors. In the univariate analysis, group A had a statistically significant better prognosis compared to group B (hazard ratio [HR] = 0.57; 95% CI 0.38–0.86; *p* = 0.007), but there was no significant difference in OS between group B and C (HR = 1.35; 95% CI 0.84–2.17; *p* = 0.217). In the multivariate analysis, males and groups with uncontrolled extracranial lesions tended to have a significantly poor prognosis (Table 2).

**Table 2:** Prognostic factors for OS in all patients (*n* = 176)

	Univariate analysis		Multivariate analysis	
	HR (95% CI)	*p* value	HR (95% CI)	*p* value
*Number of BMs*				
A (1)	0.57 (0.38–0.86)	0.007	0.59 (0.39–0.91)	0.016
B (2–4)	Ref	–	Ref	–
C (5–10)	1.35 (0.84–2.17)	0.217	1.00 (0.60–1.66)	0.997
*Age (years)*				
< 65	Ref	-	Ref	-
≥ 65	1.21 (0.80–1.81)	0.368	1.14 (0.75–1.73)	0.554
Gender				
Female	Ref	–	Ref	–
Male	1.74 (1.20–2.51)	0.003	1.71 (1.16–2.53)	0.007
*Primary site*				
Lung	1.56 (0.57–4.29)	0.386	1.08 (0.37–3.13)	0.885
GI	4.12 (1.39–12.12)	0.011	2.43 (0.77–7.64)	0.129
Breast	Ref	–	Ref	–
Others	2.26 (0.79–6.47)	0.130	1.61 (0.53–4.90)	0.401
KPS				
≥ 80	Ref	–	Ref	–
≤ 70	2.04 (1.42–2.91)	< 0.001	1.63 (1.10–2.41)	0.015
*Extracranial lesions*				
Controlled	Ref	–	Red	–
Uncontrolled	3.35(2.06–5.43)	< 0.001	2.97 (1.79–4.92)	< 0.001
*fSRT or SRS*				
SRS	Ref	–	Ref	–
fSRT	1.31 (0.91–1.87)	0.142	0.92 (0.58–1.46)	0.728
*Total PTV volume (cm*^3^)				
1 (≥ 15)	1.98 (1.25–3.19)	0.086	1.35 (0.74–2.49)	0.330
2 (≥ 4, < 15)	1.45 (0.95–2.22)	0.003	1.30 (0.81–2.08)	0.278
3 (< 4)	Ref	–	Ref	–

*BMs* Brain metastases, *GI* Gastrointestinal cancers, *KPS* Karnofsky performance status, *fSRT* Fractionated stereotactic radiotherapy, *SRS* Stereotactic radiosurgery, *PTV* Planning target volume

**Fig. 3:**
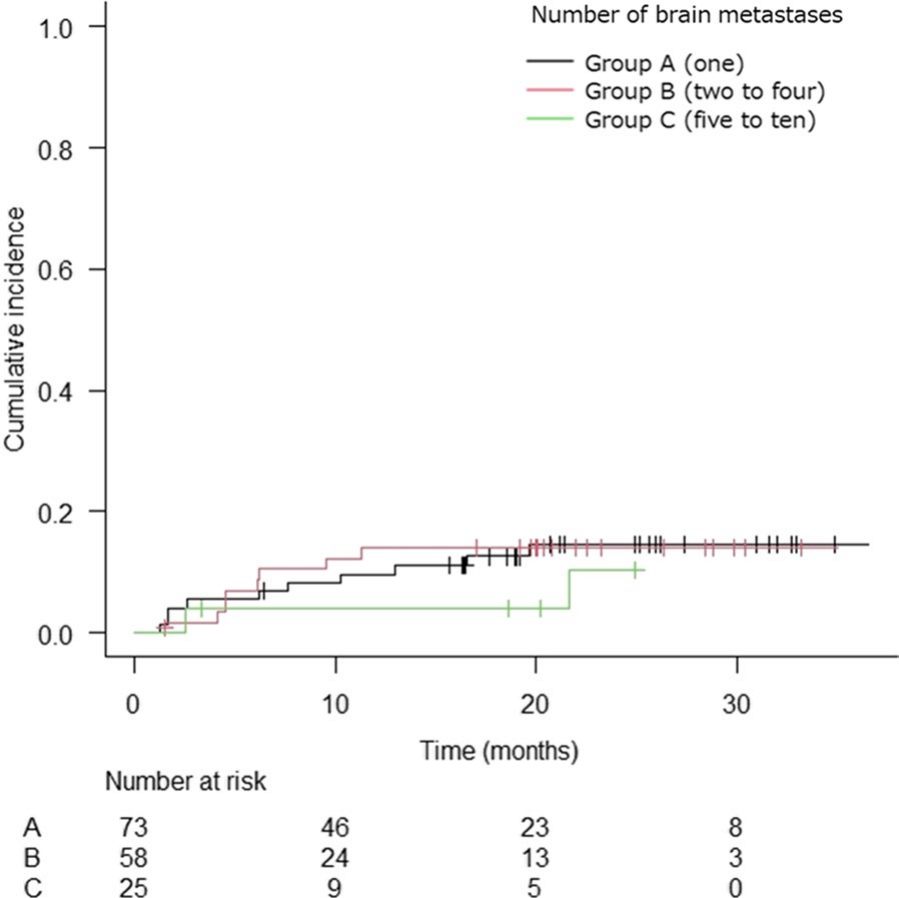
The Kaplan–Meier curve of cumulative incidence of local failure by tumor number groups (*n* = 156)

The local control rate was estimated according to tumor number groups. The cumulative incidence of local failure (LF) was comparable between tumor number groups, with cumulative LF at 1 year of 9.6%, 14.0%, and 4.0% in groups A, B, and C, respectively (Fig. 3).

In the multivariate analysis, cumulative LF was more frequent in patients with uncontrolled extracranial lesions, but the difference was not significant (Table 3).

**Table 3:** Univariate and multivariate competing risk regression analyses for local failure (*n* = 156)

	Univariate analysis		Multivariate analysis	
	HR (95% CI)	*p* value	HR (95% CI)	*p* value
*Number of BMs*				
A (1)	0.98 (0.39–2.74)	0.960	0.96 (0.30–3.04)	0.940
B (2–4)	Ref	–	Ref	–
C (5–10)	0.56 (0.12–2.59)	0.460	0.54 (0.11–2.70)	0.450
*Age (years)*				
< 65	Ref	–	Ref	–
≥ 65	0.71 (0.29–1.76)	0.460	0.70 (0.22–2.26)	0.550
Gender				
Female	Ref	–	Ref	–
Male	2.09 (0.81–5.36)	0.130	2.48 (0.80–7.66)	0.110
*Primary site*				
Lung	0.37 (0.08–1.64)	0.190	0.20 (0.03–1.18)	0.075
GI	0.40 (0.56–2.91)	0.370	0.26 (0.03–2.55)	0.250
Breast	Ref	–	Ref	–
Others	0.59 (0.11–3.06)	0.530	0.33 (0.05–2.09)	0.240
KPS				
≥ 80	Ref	–	Ref	–
≤ 70	1.60 (0.67–3.81)	0.290	1.45 (0.49–4.30)	0.510
*Extracranial lesions*				
Controlled	Ref	–	Ref	–
Uncontrolled	0.35 (0.14–0.83)	0.018	0..30 (0.10–0.93)	0.037
fSRT or SRS				
SRS	Ref	–	Ref	–
fSRT	3.45 (1.37–8.90)	0.009	1.24 (0.29–5.40)	0.770
*Total PTV volume (cm*^3^)				
1 (≥ 15)	5.50 (1.76–17.19)	0.003	4.05 (0.90–	0.068
2 (≥ 4, < 15)	1.78 (0.51–6.15)	0.360	1.47 (0.36–6.07)	0.590
3 (< 4)	Ref	–	Ref	–

*BMs* Brain metastases, *GI* Gastrointestinal cancers, *KPS* Karnofsky performance status, *fSRT* Fractionated stereotactic radiotherapy, *SRS* Stereotactic radiosurgery, *PTV* Planning target volume

**Fig. 4:**
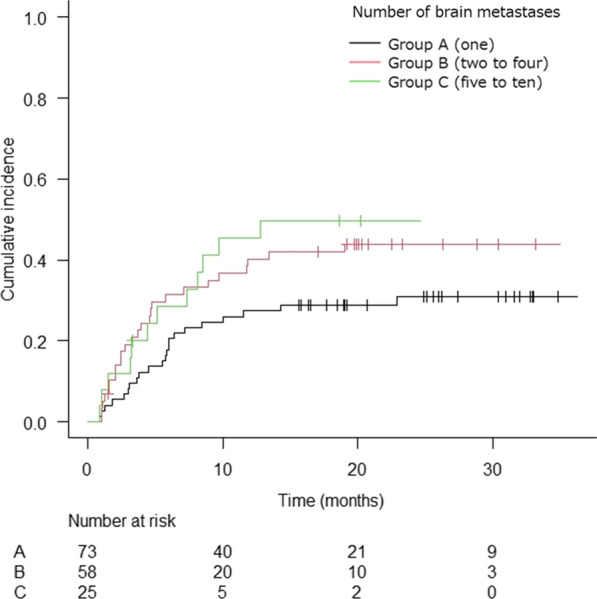
The Kaplan–Meier curve of cumulative incidence of distant intracranial failure by tumor number groups (*n* = 156)

The cumulative DIF at 1 and 2 years after treatment was analyzed by considering the competing risk. DIF occurred less frequently in Group A than Group B, and DIF was comparable between Groups B and C (Fig. 4).

In the multivariate analysis, cumulative DIF was less frequent in group A, patients with controlled extracranial lesions, and patients aged ≥ 65 years, but the difference was not significant (Table 4).

**Table 4:** Univariate and multivariate competing risk regression analyses for distant intracranial failure (*n* = 156)

	Univariate analysis		Multivariate analysis	
	HR (95% CI)	*p* Value	HR (95% CI)	*p* Value
*Number of BMs*				
A (1)	0.61 (0.34–1.08)	0.088	0.57 (0.31–1.02)	0.057
B (2–4)	Ref	–	Ref	–
C (5–10)	1.15 (0.58–2.26)	0.690	1.18 (0.53–2.60)	0.690
*Age (years)*				
< 65	Ref	–	Ref	–
≥ 65	1.21 (0.80–1.81)	0.368	0.49 (0.27–0.90)	0.020
*Gender*				
Female	Ref		Ref	–
Male	0.78 (0.47–1.30)	0.340	0.62 (0.34–1.13)	0.120
*Primary site*				
Lung	1.50 (0.37–6.12)	0.570	2.67 (0.63–11.41)	0.180
GI	1.75 (0.36–8.38)	0.490	3.41 (0.67–17.45)	0.140
Breast	Ref	–	Ref	–
Others	1.66 (0.38–7.34)	0.500	2.82 (0.63–12.67)	0.180
*KPS*				
≥ 80	Ref	–	Ref	–
≤ 70	1.00 (0.59–1.69)	0.990	1.08 (0.60–1.94)	0.800
*Extracranial lesions*				
Controlled	Ref	–	Ref	–
Uncontrolled	1.80 (0.99–3.27)	0.052	1.70 (0.94–3.09)	0.082
*fSRT or SRS*				
SRS	Ref	–	Ref	–
fSRT	0.82 (0.48–1.37)	0.440	1.04 (0.52–2.07)	0.920
*Total PTV volume (cm*^3^)				
1 (≥ 15)	0.83 (0.43–1.60)	0.580	0.70 (0.30–1.62)	0.410
2 (≥ 4, < 15)	0.64 (0.35–1.16)	0.140	0.59 (0.30–1.16)	0.130
3 (< 4)	Ref	–	Ref	–

*BMs* Brain metastases, *GI* Gastrointestinal cancers, *KPS* Karnofsky performance status, *fSRT* Fractionated stereotactic radiotherapy, *SRS* Stereotactic radiosurgery, *PTV* Planning target volume

### Toxicity

**Fig. 5:**
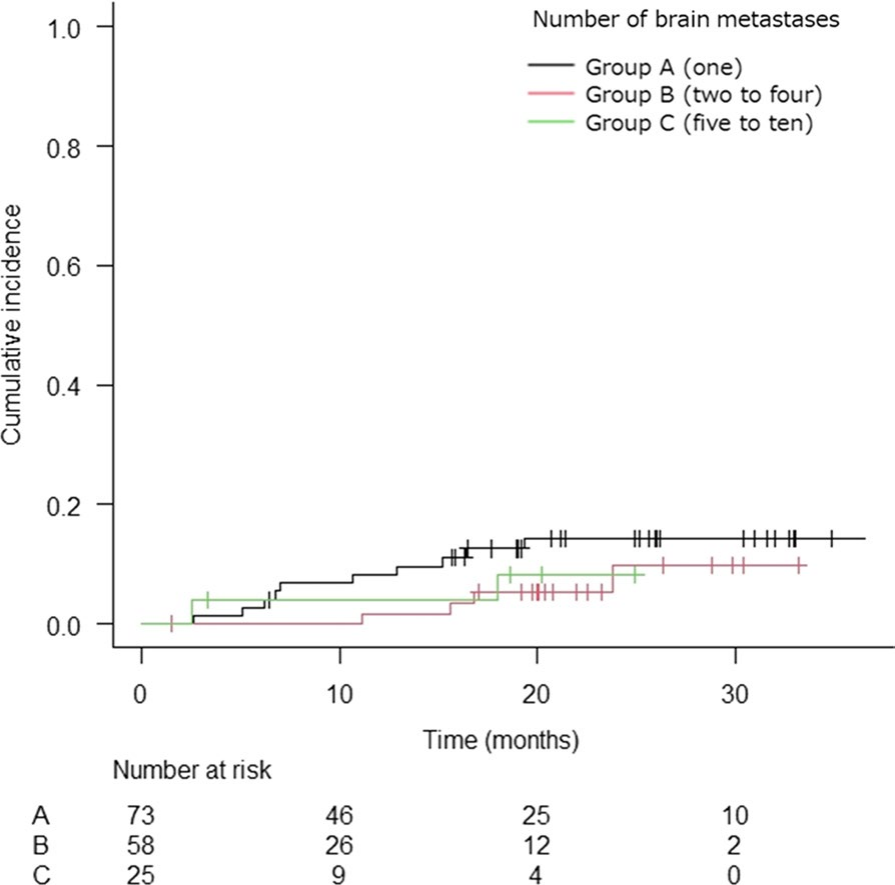
Kaplan–Meier curve of cumulative incidence of grade 2 or higher RN by tumor number (*n* = 156)*.* RN, radiation necrosis

In 156 patients that could be evaluated by imaging, the cumulative incidence of grade 2 or higher RN after treatment was observed in 21 patients (Fig. 5); RN of grade 2 were observed in 14 patients (9.0%) and grade 3 in 7 patients (4.5%). In the univariate and multivariate competing risk regression analyses of grade 2 or higher RN, none of the clinical factors seemed to have been associated with RN (Table 5).

**Table 5:** Univariate and multivariate competing risk regression analyses of Grade 2 or higher radiation necrosis (RN) (*n* = 156)

	Univariate analysis		Multivariate analysis	
	HR (95% CI)	*p* Value	HR (95% CI)	*p* Value
*Number of BMs*				
Group A	2.10 (0.67–6.60)	0.200	1.72 (0.53–5.60)	0.370
Group B	Ref	–	Ref	–
Group C	1.21 (0.22–6.52)	0.820	1.13 (0.18–7.15)	0.900
*Age (years)*				
< 65	Ref	–	Ref	–
≥ 65	0.81 (0.28–2.35)	0.700	0.79 (0.24–2.57)	0.690
*Gender*				
Female	Ref	–	Ref	–
Male	1.13 (0.42–2.98)	0.810	0.91 (0.32–2.59)	0.870
*Primary site*				
Lung	Ref	–	Ref	–
GI	0.44 (0.06–3.44)	0.430	0.57 (0.07–4.91)	0.610
Breast	N.A	N.A	N.A	N.A
Others	0.50 (0.11–2.16)	0.350	0.53 (0.11–2.61)	0.440
*KPS*				
≥ 80	Ref	–	Ref	–
≤ 70	0.48 (0.16–1.48)	0.200	0.52 (0.12–2.21)	0.370
*Extracranial lesions*				
Controlled	Ref	–	Ref	–
Uncontrolled	0.33 (0.12–0.87)	0.025	0.44 (0.15–1.31)	0.140
*fSRT or SRS*				
SRS	Ref	–	Ref	–
fSRT	1.34 (0.51–3.54)	0.560	1.35 (0.35–5.16)	0.670
*Total PTV volume (cm*^3^)				
1 (≥ 15)	1.10 (0.27–4.59)	0.890	1.57 (0.24–10.33)	0.640
2 (≥ 4, < 15)	1.86 (0.61–5.64)	0.270	2.00 (0.51–	0.320
3 (< 4)	Ref	–	7.91)Ref	–

*RN* Radiation necrosis, *BMs* Brain metastases, *GI* Gastrointestinal cancers, *KPS* Karnofsky performance status, *fSRT* Fractionated stereotactic radiotherapy, *SRS* Stereotactic radiosurgery, *PTV* Planning target volume

## Discussion

In this study, patients with one BM (group A) had better OS than those with 2–4 BMs (group B) and 5–10 BMs (group C), but there was no significant difference in OS between groups B and C.

A prospective study of gamma knife SRS for BMs by Yamamoto et al. [2] (JLGK 0901) showed that the OS of patients with 5–10 BMs was non-inferior to that of patients with 2–4 BMs. The median total volume of the included lesions was 2.84 cm^3^. In a multicenter retrospective study of SRS with a gamma knife, Hugh et al. [3] found that the OS of patients with 5–15 BMs was comparable to that of those with 2–4 BMs. This study did not mention tumor volume; however, a similar single-center study by Hugh et al. [17] reported that the median tumor volume was 1.99 cm^3^. Meanwhile, the median total PTV in our study was 6.03 cm^3^, and patients with a total PTV of ≥ 4 cm^3^ accounted for 63% of all patients. In the multivariate analysis, total PTV was not a prognostic determinant, and in linac-based fSRT and SRS for up to 10 BMs, PTV seemed insignificant for OS.

However, in contrast to our study, several studies, including those by Likhacheva et al. [4], Routman et al. [7], and Alongi et al. [9], reported that total tumor volume is a prognostic factor for OS in fSRT and SRS for BMs. Likhacheva et al. [4] showed that LC for all tumors at 1 year was 94.6%, and LC at 1 year for tumors larger than 3 cm [3] was not less than 80%. However, given that the median OS for all patients was 11 months and that the total tumor volume was a significant prognostic factor for LC in their study, LC may be influenced by competing risks (e.g., mortality). The reason why total tumor volume was more important for OS than the number of BM might be that LC for large BMs was relatively poor.

In a multivariate analysis of OS, Routman et al. [7] showed that patients with a total tumor volume of 10 cm^3^ or more had a significantly poorer prognosis than those with a total tumor volume of 5 cm^3^ or less and that the number of BMs was not a significant prognostic factor. However, they did not present an analysis for LC. Since the number of BM accounted for only 4.5% of the variance in tumor volume and total tumor volume was similar for 2–4 BMs and 5 or more BMs in their study, it seems that one or two large BMs can account for a large portion of the total tumor volume. Again, the reason why total tumor volume was more important for OS than the number of BMs may be relatively poor LC for large BMs.

Although lesions with a diameter ≥ 30 mm were excluded in a study by Alongi et al. [9], they reported outcomes of mono-isocentric linac-based fSRT and SRS for 1–22 BMs; they showed tumor volume-defined OS rather than number of BMs. Alongi et al. [9] found that tumor volume (GTV ≤ 0.2 cm^3^) was also a significant prognostic factor in LC; considering that about half of deaths were neurological death, it was inferred that local recurrence affected OS.

In our study, LC tended to be somewhat worse in patients with a higher total tumor volume, but total tumor volume was not a significant prognostic factor for either OS or LC. The total tumor volume was not a determinant of LC, possibly because we opted to apply fSRT for large lesions; Masucci [18] reported the usefulness of fSRT in achieving good local control while minimizing the frequency of RN. The LC and RN shown in our study were comparable to those in a previous review by Masucci [18], and we believe that appropriate dose fractionation was selected at our institution.

Unlike reports by Yamamoto et al. [2] and Hugh et al. [3], factors such as age > 65 years and primary site were not significantly different in the multivariate analysis of OS in this study. Given that the percentage of patients with RPA class 1 was 6% in this study compared to 28% in the study by Yamamoto et al. [2] and that the percentage of patients with progressive extracranial lesions was 78% in this study compared to 56% in the report by Hugh et al. [3], it is likely that the proportion of advanced cases was higher in our study than in previous reports. In the study by Yamamoto et al. [2], the median survival was 10.8 months for patients with 2–4 and 5–10 BMs, and Hugh et al. [3] reported a median survival of 9.5 months for 2–4 BMs and 7.5 months for 5–15 BMs. In this study, the OS of patients other than those with one BM was somewhat worse than in these two studies [2, 3], which may have been due to a higher proportion of advanced cases. Therefore, age and primary site may not have been significant prognostic factors in the multivariate analysis of OS in our study. In addition, only 5% of the patients in this study had breast cancer, whereas the proportion of patients with breast cancer was 10% in the studies by Yamamoto et al. [2] and 14% in Hugh et al. [3] The fact that the proportion of patients with breast cancer, which is generally regarded to have a favorable prognosis, was small in this study may have contributed to the slightly inferior OS of patients other than those with a solitary tumor compared with previous reports, [2, 3] and may also have contributed to the result that the primary sites were not found to be a significant factor in OS in this study.

Our study showed that patients with uncontrolled extracranial lesions had significantly poorer OS, which was consistent with the reports of Yamamoto et al. [2] and Hugh et al. [3] Brain metastases velocity (BMV) is known as the number of new metastases between the first SRS and the next SRS divided by time and is considered to reflect the overall disease status. [19] Although we did not measure BMV in this study, since patients with uncontrolled extracranial lesions tended to have more frequent DIF, such patients may have had higher BMV, and BMV may be a prognostic factor for OS.

This study had several limitations. As a retrospective study, the patient background in our study may differ from that in previous reports due to the relatively small number of breast cancer patients, as mentioned above. Although our study did not include systemic therapy as a prognostic factor in the analysis because the primary histology varied, we conducted a post hoc analysis with systemic treatment added as a factor. The results of the main analysis remained unchanged, but the OS was significantly longer in patients who received systemic treatment after fSRT or SRS in both univariate (HR = 1.72; 95% CI 1.20–2.47; *p* = 0.003) and multivariate analysis (HR = 2.24; 95% CI 1.48–3.39; *p* < 0.001). Systemic therapy was not a significant factor for LC or RN in the multivariate analysis (data not shown). The poor prognosis of the group that did not receive systemic therapy may have influenced the results. This suggests the need for further investigation in future prospective trials to determine the influence of systemic treatment on outcomes of fSRT and SRS for BMs in patients with various malignant backgrounds. Regarding RN, our study did not analyze the dose-volume relationship for the normal brain and RN occurrence because cases treated with either various dose fractions or for multiple lesions during the same treatment course were included. The optimal dose constraints for the normal brain, especially when treating multiple lesions, will be an issue for future study. Also, in this study, we did not compare the outcomes of patients with more than four BMs receiving fSRT or SRS with those receiving WBRT; we will have to wait for the report by Roberge et al., a phase III trial of stereotactic radiosurgery compared with WBRT for 5–15 BMs [20]. Lastly, the intervals between MRI scans during follow-up were different in some cases. The timing of local recurrence, RN, and DIF may have deviated from the actual timing, partially explaining why DIF was less frequent in older patients. Nevertheless, most of the patients were followed up at our institution (97.2%). Our finding that there was no significant difference in OS between patients with 2–4 BMs and those with 5–10 BMs seems plausible.

## Conclusions

There was no significant difference in OS between patients with 5–10 BMs and those with 2–4 BMs in linac-based fSRT and SRS, even if large BMs were included. It seems reasonable to use linac-based fSRT and SRS in patients with 5–10 BMs.

## Data Availability

Research data were stored in an institutional repository. Collected data are not planned to be shared; however, we are open to the research questions asked by other researchers.

## Abbreviations

BMs: Brain metastases
WBRT: Whole-brain radiotherapy
OS: Overall survival
LC: Local control
DIF: Distant intracranial failure
RN: Radiation necrosis
HR: Hazard ratio
SRS: Stereotactic radiosurgery
fSRT: Fractionated stereotactic radiotherapy
GI: Gastrointestinal
RPA: Recursive partitioning analysis
KPS: Karnofsky performance status
MRI: Magnetic resonance imaging
CT: Computed tomography
GTV: Gross tumor volume
PTV: Planning target volume
rCBV: Relative cerebral blood volume ratio
LF: Local failure
BMV: Brain metastases velocity
