# Correction: Feasibility of linac-based fractionated stereotactic radiotherapy and stereotactic radiosurgery for patients with up to ten brain metastases

**DOI:** 10.1186/s13014-023-02206-7

**Published:** 2023-03-03

**Authors:** Masanori Hirata, Kazuaki Yasui, Naofumi Oota, Hirofumi Ogawa, Tsuyoshi Onoe, Sayo Maki, Yusuke Ito, Kenji Hayashi, Hirofumi Asakura, Shigeyuki Murayama, Koichi Mitsuya, Shoichi Deguchi, Katsumasa Nakamura, Nakamasa Hayashi, Tetsuo Nishimura, Hideyuki Harada

**Affiliations:** 1 Radiation and Proton Therapy Center, Shizuoka Cancer Center, 1007 Shimonagakubo, Nagaizumi-Cho, Sunto-Gun, Shizuoka, 411-8777 Japan; 2 Division of Neurosurgery, Shizuoka Cancer Center, Shizuoka, Japan; 3 Department of Radiation Oncology, Hamamatsu University Hospital, Shizuoka, Japan

**Correction to: Radiation Oncology (2022) 17:213** 10.1186/s13014-022-02185-1

After publication of this article [1], the authors reported that (1) In Table 1, first column, under ‘RPA’, 3 lines should start with ‘1, 2, 3’, respectively (instead of ‘12, 3, blank’); (2) In 5 spots the value ‘x cm [3]’ should have read ‘x cm^3^’.

The original article [1] has been corrected.
